# CRISPRware: a software package for contextual gRNA library design

**DOI:** 10.1186/s12864-025-11775-8

**Published:** 2025-07-01

**Authors:** Eric Malekos, Christy Montano, Susan Carpenter

**Affiliations:** 1 Department of Biomolecular Engineering, University of California Santa Cruz, Santa Cruz, CA USA; 2 Department of Molecular, Cell, and Developmental Biology, University of California Santa Cruz, Santa Cruz, CA USA

**Keywords:** CRISPR screen, Guide RNA, Next generation sequencing

## Abstract

We present CRISPRware, an efficient method for generating guide RNA (gRNA) libraries against transcribed, translated, and noncoding regions. CRISPRware leverages next-generation sequencing data to design context-specific gRNAs and can account for genetic variation, which allows allele-specific guide design on a genome-wide scale. As a demonstration of use and to create a publicly available resource, we use CRISPRware to identify and score gRNAs against coding sequences in six model organisms for Cas9 and Cas12A and host these in a publicly available session on the UCSC Genome Browser.

**Supplementary Information:**

The online version contains supplementary material available at 10.1186/s12864-025-11775-8.

## Background

CRISPR-Cas systems are widely used for both targeted experiments and genome-wide perturbation screens. In both cases, a successful experiment begins with choosing guide RNAs (gRNAs) complementary to the genomic target region (or protospacer) and predicted to have high on-target activity and low off-target activity. While significant attention has been paid to predicting on-target [1–7] and off-target [1, 8–10] activity for a predetermined set of gRNAs, there are few robust methods for selecting gRNAs in a high throughput fashion at genomic scale. Existing tools for genome-scale library design include the CRISPOR [11] and the Guidescan2 [8] web portals, both of which are limited to pre-constructed libraries against a limited number of genomes. The Broad Institute’s CRISPick, also a web portal, can be used to construct libraries targeting human, mouse, and rat genes but has a limit of 500 target sites (genes or transcripts) per submission. Beyond the limited number of genomes and gene annotations available in these tools, web portals broadly suffer from a lack of flexibility and customizability for users. Locally installed tools for gRNA selection at scale are available [9, 12, 13] (Table 1) and offer more features and the ability to work with any genome and transcriptome. However, these tools often rely on dated on-target scoring criteria and insensitive off-target criteria that may result in suboptimal gRNA libraries.

**Table 1:** Open source tools for flexible, high throughput CRISPR library design

Tool	On-target methods	Off-target search
CRISPR Library Design [13]	Ruleset 1 (Doench et al., 2014) [5], Spacer Scoring for CRISPR (SSC) (Xu et al. 2015) [14]	Bowtie [15], Bowtie2 [16], BLAST [17]
FlashFry [9]	Ruleset 1 (Doench et al., 2014) [5], CRISPRscan (Moreno-Mateos et al. 2015) [7]	FlashFry [9]
multicrispr [12]	Ruleset 2 (Doench et al., 2016) [1]	Bowtie [15], Aho–Corasick [18]*
CRISPRware	Ruleset 3 (DeWeirdt et al., 2022) [3]^#^	Guidescan2 [8]

^*^Like Guidescan2 and FlashFry, this algorithm guarantees finding all off-target sites but “is prohibitively slow for large- and even medium-scale applications.”

^#^CRISPRware facilitates scoring with crisprVerse, allowing additional on-target methods, including DeepHF and DeepSpCas9 for Cas9 and DeepCpf1 and enPAM + GB for Cas12A

Underperformant on-target (or cleavage-efficiency) calculations can result in lower gene knockout and increase the likelihood of false-negative results in CRISPR screen experiments. Earlier on-target scoring methods relied on smaller datasets and simpler machine learning algorithms and have been outperformed by more sophisticated recent approaches. For an illustrative example, consider the evolution of the CRISPR Ruleset series. Ruleset 1 [5] was trained on 1,841 gRNA sequences and used a single dataset. Ruleset 2 (Azimuth) [1] on 4,390 sequences across two datasets. Ruleset 3 [3] was trained on 46,526 sequences across seven datasets. Importantly, Ruleset 3 added predictive features, accounting for the presence of poly-T sequences (a U6 termination signal) and the folding energy of the full single guide RNA sequence, which resulted in stronger predictive performance compared to previous models. Many additional on-target scoring methods are used beyond the Ruleset series**.** Notably, these include deep learning methods that require little or no explicit feature selection, instead making predictions based on information extracted from the protospacer and its surrounding sequence. Among these, DeepSpCas9 [4] and DeepHF [2] have demonstrated high accuracy across several cell lines [19]. Noting the oftentimes poor correlation among on-target prediction methods, some have proposed ensemble methods—generating a new score by combining the outputs of multiple tools [19–21]. Sometimes, this has taken the form of applying a machine learning model to outputs of on-target tools, but surprisingly, simply taking the arithmetic mean of outputs from multiple on-target scores has been shown to match and outperform the individual predictions in tested cases [19]. Thus, ensemble methods potentially offer improved prediction without requiring the development of complex new models, but due to useability challenges and the complexity of maintaining different programmatic environments for various on-target tools, it can be challenging to obtain multiple on-target scores for a set of gRNAs in the first place. CrisprVerse [22], a Bioconductor package, solves this problem by providing a single interface for scoring gRNAs with nine popular methods, including DeepCpf1 [23] and enPAM+GB [24] for the Cas12A (Cpf1) system**.**

Using optimal off-target tools is also an essential aspect of screen design. Failing to account for off-target effects may result in unexpected double-stranded breaks in active CRISPR experiments or silencing of distal genetic elements in CRISPRi experiments. Due to permissive binding of the gRNA, it is important to consider not only exact matches but also similar sequences—typically allowing up to three mismatches in the protospacer. The first step in calculating off-target scores is to enumerate all sequences in the genome that share sequence similarity to a given protospacer. This is a computationally expensive problem and is the rate-limiting step in genome-scale CRISPR screen design. Many design tools address this by repurposing short-read alignment tools such as Bowtie [15, 16] and BLAST [17], but these tools are optimized for finding *optimal matches* as opposed to finding *all possible near-matches*. Consequently, these aligners are known to miss off-targets, especially when allowing mismatches [1, 12, 25]. To address this issue, tailor-made off-target algorithms have been developed and are discussed by Li et al [25]. Among the tools that guarantee all identical and mismatched sequences are FlashFry [9] and Guidescan2 [8]. Guidescan2 off-target scoring has the benefit of being distributed as a Bioconda package and has been extensively tested and benchmarked for experimentally relevant parameters and cut-offs [8, 10, 26, 27].

Beyond missing the inclusion of the most robust modern scoring methods, there is no high throughput tool that allows users to easily use next-generation sequencing (NGS) data from genomic and transcriptomic sources to design gRNA libraries for specific cell types or experimental conditions. These challenges and shortfalls are addressed in CRISPRware, a software package that combines validated scoring techniques with rational design principles to allow users to efficiently generate libraries for single genes or entire genomes.

## Results

**Fig. 1:**
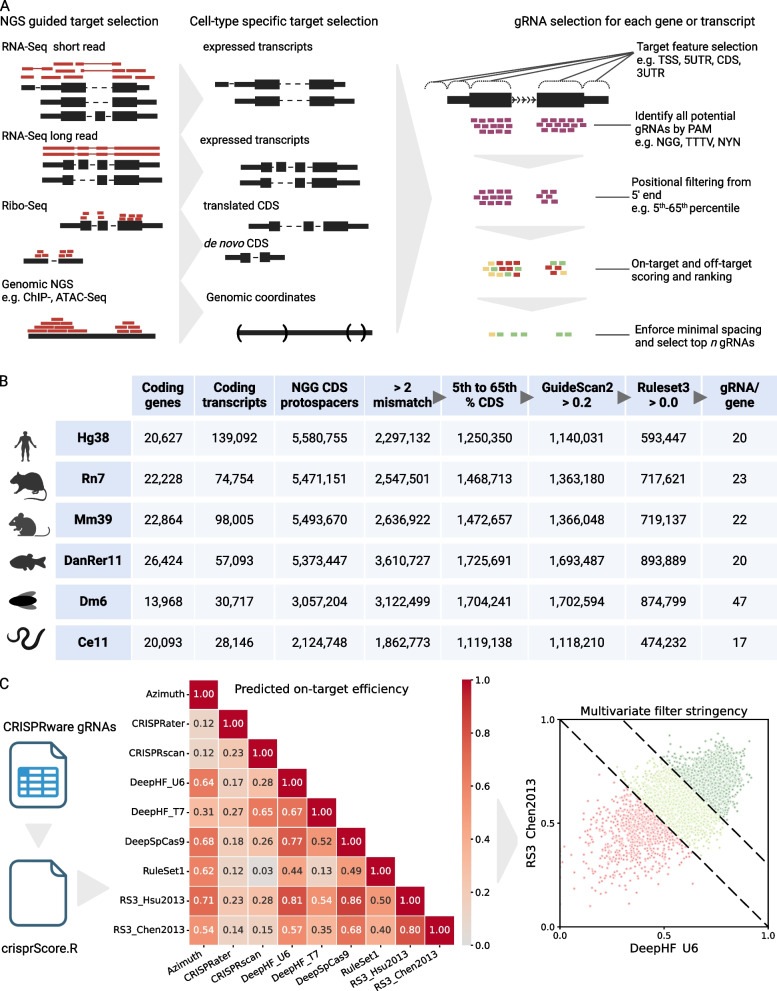
**A** Overview of CRISPRware gRNA selection pipeline. Left, CRISPRware takes a genomic fasta and a target file (GTF/GFF or BED). Left and center, optionally, a variety of processed NGS data can be used to inform target selection. Right, CRISPRware implements methods for on-target and off-target scoring and established practices for selecting a top set of gRNAs for genes and transcripts. **B** Demonstration of CRISPRware applied on model genomes with NCBI RefSeq gene annotations. After identifying protospacers (column 3), the standard CRISPRware filtering pipeline is applied, with subsequent columns applying all previous filters. 5th-65th percentile CDS filtering is based on previous reporting. The final column reports the median gRNAs available for each gene, one of the summary readouts provided at each filtering stage. **C** Left, CRISPRware is packaged with Ruleset 3 (RS3) but has built-in interoperability with the crisprScore module of crisprVerse, which allows many additional scoring methods. Center, Spearman correlation matrix of crisprScore Cas9 techniques targeting Hg38 coding sequences. Right, RS3 scoring with Chen2013 tracrRNA versus DeepHF scoring with U6 promoter after applying max–min normalization to both methods. 5,000 randomly selected Hg38 CDS-targeting guides are displayed

CRISPRware is a Python software package that can use various processed NGS data types to generate gRNA libraries. The workflow starts by defining genomic targets of interest (e.g. gene coding sequences or transcription factor binding sites), determining available protospacers in target regions, scoring the putative protospacer-targeting gRNAs for on-target and off-target activity, and returning a ranked list of gRNAs for each target (Fig. 1A). Off-target activity is determined by GuideScan2 and on-target scoring (or cleavage efficiency) is determined by Ruleset 3 (Table 1). To demonstrate the core functionality of CRISPRware, we document all available NGG (Cas9) and TTTV (Cas12A or Cpf1) protospacers in the coding sequences (CDS) of NCBI RefSeq genes for the human, mouse, rat, zebrafish, fruit fly, and nematode (Fig. 1B, Supp Fig 1A, Supp Tables 1–12). On-target scoring has been shown to benefit from an ensemble approach combining multiple scoring methods [19–21]. Therefore, we made CRISPRware compatible with many other on-target scoring methods via the crisprVerse [22] Bioconductor package (Fig. 1C). We make gRNAs with scores from all available tools accessible as tracks on the UCSC Genome Browser [28] for these six organisms (Availability of data and materials section). To facilitate the widespread use of CRISPRware, we implement a batch-size parameter that allows users to score gRNAs efficiently even on personal computers with limited resources (Supp Fig 1B).

**Fig. 2:**
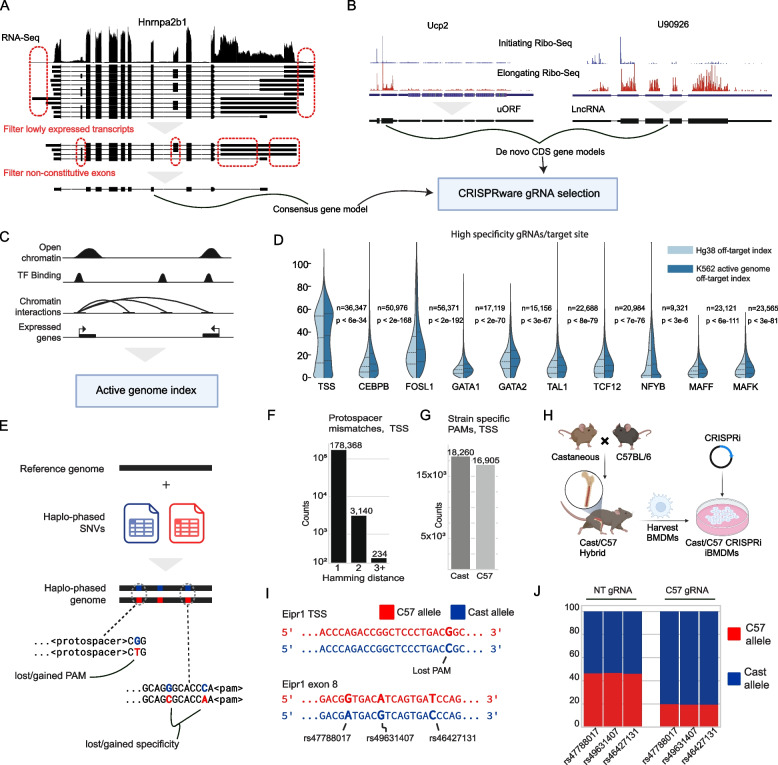
**A** Demonstration of RNA-Seq guided target selection. Top, RNA-Seq at Hnrnpa2b1 locus in mouse bone marrow-derived macrophages (BMDMs). Middle, remaining transcripts after expression filtering. Bottom, consensus gene models are constructed from shared isoform regions. **B** Demonstration of Ribo-Seq guided target selection. De novo gene models are built from noncoding regions with predicted translated open reading frames (**C**) Schematic of CRISPRware cell type-specific off-target database construction from processed genomic NGS data. **D** Availability of gRNAs with high specificity (GuideScan2 score > 0.2) against either the entire Hg38 genome or the active portions of the genome in K562. See Methods for construction of K562 active genome. **E** Schematic of CRISPRware allele-specific targeting. **F**, **G** Mismatches in the protospacer sequence between C57BL/6 and Castaneous in TSS and PAMs specific to either C57BL/6 or Castaneous appearing in the TSS. TSS-targeting is defined as a ± 300 bp window around a Gencode vM34 TSS. **H** Generation of C57BL/6 × Castaneous CRISPRi BMDMs. **I** Top, a PAM site in the TSS of Eipr1 in C57BL/6 is lost in Castaneous. Bottom, three SNPs in exon 8 can be used to discern allele-specific Eipr1 expression. **J** Relative expression of Eipr1 from Amplicon-Seq, with either a non-targeting gRNA or a C57 targeting gRNA

While the number of annotated protein-coding genes has stabilized, novel protein-coding isoforms, transcription start sites, noncoding genes, and genes that encode small peptides continue to be discovered (Sup Fig. 2A-D) [29]. The isoform expression, TSS usage, and translation of noncanonical reading frames are cell-type and context-specific. These observations argue against the use of static CRISPR gRNA libraries and towards contextual library design. For instance, CRISPRware can use RNA-Seq transcript per million (TPM) data from popular long-read [30, 31] and short-read tools [32, 33] to filter based on isoform expression. Depending on the desired complexity of the gRNA library, the user can target all isoforms above some defined TPM cut-off or generate new gene models with a single isoform-per-gene (Fig. 2A). In testing various stringency cut-offs with cell-line specific RNA-Seq, we found that a consensus gene model, composed of shared exons and CDS features of all isoform variants for a given gene, could almost always be constructed (Sup Fig. 2E–F). Using such models solves the optimization problem of choosing the fewest gRNAs with the highest likelihood of knocking out gene function.

CRISPRware can also use processed Ribo-Seq (or Ribosome footprinting) data to target translated canonical CDSs and novel coding ORFs in regions annotated as noncoding. Recently, CRISPR screen experiments have been used to characterize these novel ORFs and the peptides they encode [34, 35]. However, targeting these ORFs is not natively supported by current CRISPR screen design tools. CRISPRware addresses this unmet need by generating de novo gene model annotations from Ribo-Seq analysis (Figs. 1A and 2B) provided by either of two widely used tools [36, 37], which can be used as input to the CRISPRware pipeline.

CRISPRware supports targeting noncoding areas such as promoter and enhancer regions for CRISPR inhibition (CRISPRi) and activation (CRISPRa) experiments. Successful targeting of noncoding regions is known to be highly dependent on the chromatin state of the target site [27, 38, 39] and the protospacer position relative to the transcription start site (TSS) [27]. Notably, CRISPRware can combine processed NGS data to leverage these known properties. For example, using RNA-Seq data to determine highly active TSS sites along with either signal tracks (bigwig) or called peaks (BED) from assays against markers of active chromatin (e.g. ATAC-Seq, DNase-Seq, ChIP-Seq, etc.) to target the most accessible window in proximity to the TSS.

One challenge researchers may face in screen design is a lack of suitable gRNAs, especially when adequate off-target filtering is applied [26]. However, based on the observation that CRISPR systems are highly dependent on DNA accessibility, we developed a wrapper for GuideScan2 that can index the active portion of the genome (Fig. 2C). We demonstrate that the number of targets for which suitable gRNAs can be found for gene TSSs and various transcription factor binding sites is substantially increased for the K562 cell line with this approach (Fig. 2D, Supp Fig. 3 A), even when the active genome is liberally defined (see Methods).

We also accounted for gRNA selection in the context of genetic variation and allele-specific targeting (Fig. 2E). We generated a haplo-phased genome by lifting SNPs from the Castaneous (Cast) strain from the Mouse Genome Informatics [40] group onto mouse reference genome Mm39 (C57BL/6 [C57] strain) with a CRISPRware script (see Methods). CRISPRware was used to identify protospacers with mismatches between the two strains and lost or gained PAMs in CDSs and TSSs (Fig. 2F, G Supp Fig. 4A-F). We generated immortalized bone marrow-derived macrophages from a C57-Cast hybrid F1 mouse line and transduced it with CRISPRi machinery (Fig. 2H). We used CRISPRware to design guides using RNA-Seq and ATAC-Seq from C57 and knocked down Eipr1. We sequenced the Eipr1 RNA and saw that targeting C57 Eipr1 TSS reduced expression from the C57 allele relative to the Cast allele where the PAM site was absent (Fig. 2I, J).

## Conclusion

In summary, we present a flexible CRISPR gRNA design tool that can efficiently generate gRNAs at single gene or genome-wide scale. We demonstrate its utility by determining CDS-targeting gRNAs across six species, score them with multiple on-target tools via crisprVerse, filter out those with low specificity, and release hosted UCSC Genome Browser hubs to facilitate widespread usage (see Availability of data and materials) (Supp Tables 1–12). Including the latest scoring criteria in a high-throughput library design tool should improve gRNA selection relative to previously released tools based on updated on-target and off-target scoring algorithms (Table 1). CRISPRware’s default Guidescan2 settings comport with recommendations presented in a detailed study of off-target effects [26], but also allow users to modulate the settings to increase or decrease stringency. The landscape of on-target scoring tools is more complex than off-target scoring. The available tools use a variety of machine learning algorithms, from simple linear regression models [7] to convolutional neural networks [2, 4], and use diverse measurements, including gRNA dropout [3], flow cytometry [5], and insertion-deletion frequency [2], to measure efficiency. The tools are also limited in the number of unique cell types and target sites they are trained on, which can result in overfitting the models to specific contexts. This may be the reason that ensemble methods, including simply averaging the scores of high-performing methods [19], can outperform individual methods—the biases inherent to each are effectively averaged out. For this reason, we include a parameter in the final module in the CRISPRware pipeline to calculate a weighted average of the user’s selected score columns (setting all score weights to “1” yields the standard mean across scores). We anticipate that allowing straightforward aggregation of scores across on-target methods at genome-scale will allow further validation of on-target efficacy in future studies in varied contexts.

Most of the on-target scoring methods discussed above use the protospacer sequence alone or a slightly expanded window that includes the PAM for prediction purposes. While the features extracted from these small sequences have demonstrated predictive power, they cannot account for other target features that have been rationally deduced and empirically demonstrated to be important for efficient CRISPR-Cas activity. These features include targeting near the 5’ end of the coding sequence [1, 41], targeting shared exons across isoforms [13, 41], and the openness of chromatin at the target site [27]. While growth in the number of documented isoforms of coding and noncoding genes (Supp Fig. 2A-D) complicates the process of optimally targeting genes, transcript quantification via Salmon and Kallisto makes it simple to determine the dominant isoform(s) in any experimental context with available RNA-Seq. CRISPRware’s ability to integrate this information and generate consensus gene models (Fig. 2A) provides a rational solution to this problem that is missing in static libraries that do not consider transcriptomic complexity.

Like alternative splicing, chromatin openness, as determined by ATAC-Seq, DNase I hypersensitivity (DHS), and similar assays, can differ significantly between cell types. Moreover, the positive effect of open chromatin with active-Cas9, dead-Cas9 (CRISPRi/a), and Cas12 A on gRNA efficiency is well supported [23, 38, 39, 42, 43], with a recent study on dozens of CRISPRi screens demonstrating the proximity to DHS peaks is highly predictive of knockdown independent of protospacer sequence [27]. Histone modifications add another layer of complexity and strengthen the case for using cell-type specific NGS data for context-specific library design over one-size-fits-all static libraries. Our understanding of the impact of histone modifications on gRNA on-target efficacy is incomplete; it has been demonstrated that CRISPRi is effective when targeted against euchromatin (marked by, e.g. H3K27ac, H3K4me3) and ineffective when targeted against heterochromatin (H3K9me3, H3K2me3) [39]. CRISPRa has been shown to be highly active when targeting bivalent chromatin with both H3K4me3 and H3K27me3 marks, indicating genes that are silent but poised for transcription [44], and which are important in diverse cellular contexts, including differentiation [44] and immune activation [45]. Thanks to the work of consortiums like Encode [46], there are databases of thousands of processed NGS datasets from diverse cell types. CRISPRware simplifies the process of choosing guides that target these relevant features at loci of interest, and this information can be combined with RNA-Seq to, for instance, target open chromatin in the promoter regions of expressed isoforms.

A further feature of CRISPRware is the ability to incorporate SNP data in gRNA design. CRISPRware can make use of SNP data in two ways. Firstly, it can filter out protospacers that include SNPs, which might otherwise reduce or ablate gRNA efficiency at those alleles. Alternatively, given a set of haplo-phased SNPs, CRISPRware can simulate searches against a diploid genome and return allele-specific gRNAs. This may facilitate future studies of cis-regulatory interactions, haplo-sufficiency, and dominant negative mutations.

## Methods

CRISPRware minimally requires a genome in fasta format and either a GTF/GFF gene annotation or BED-style interval file to find and score protospacers of interest. If run, for example, with the human genome build hg38 and gencode.47.annotation.gtf with default settings, all NGG-protospacers with cut-site intersecting any Gencode annotated exon will be discovered and scored. As described below, processed NGS data can be used to create targeted libraries.

### RNA-Seq guided preprocessing

Several scripts exist to preprocess NGS data before determining gRNAs. The module preprocess_annotation takes processed RNA-Seq TPMs from Kallisto/Salmon (short-read) or FLAIR/Mandalorian (long-read) from one or more samples along with the GTF/GFF gene annotation. If multiple samples are passed, max, min, median, and mean TPM values for each transcript are determined, and the user can supply minimum cut-offs for any combination of these to filter out lowly expressed isoforms. All detected isoforms (TPM > 0) are kept by default. The user can also set an integer flag “–-top-n <n> ”; which will filter out all but the <n> most highly expressed isoform for each gene. 

Following filtering, a filtered GTF is created, along with four optional GTF files: *shortest*, *longest*, *metagene*, and *consensus*. These four GTFs will have a single isoform model for a given gene. *Shortest* and *longest* retain the shortest and longest isoform, respectively. *Metagene* constructs a gene model consisting of all exons and coding sequences (CDSs) from all isoforms for a given gene, similar to a union operation. *Consensus* constructs a gene model in which shared or overlapping exons and CDSs from isoforms from a given gene are combined such that exon and CDS entries in the final gene model appear in all other isoforms, similar to an intersection operation. By design, the *shortest*, *longest*, and *metagene* options generate output gene models for all input genes. *Consensus* models cannot always be generated (e.g. a protein-coding gene in which there are no common CDS entries, Supp Fig. 2E, F).

Optionally, two BED files are generated for each new GTF, a transcription start site (TSS) and a transcription end site (TES) file, which generate coordinates around the TSS and TES in a user-defined window (default: ± 250 bp).

### Ribo-Seq guided preprocessing

Ribo-Seq is a technique that allows researchers to evaluate the coding potential of canonical and noncanonical open reading frames (ORFs) by sequencing ribosome-protected fragments. Ribo-Seq reads are mapped to a transcriptome, and statistical analyses are performed to infer coding regions. CRISPRware can take ORFs called from Ribo-Seq and generate new GTFs with de novo CDS entries. Currently, GTFs can be generated from the output of either Probabilistic inference of codon activities by an EM algorithm (PRICE) or Ribo TIS Hunter (Ribo-TISH), two widely used Ribo-Seq processing tools.

### ATACSeq, DNASESeq, ChIPSeq, and other genomic preprocessing

Targeting noncoding elements can be guided by any NGS data that yields BED coordinate files. A helper script, bigwig_to_signalwindow.py, can also take a BED and BigWig signal file and return the window in each BED entry with the highest mean signal. For example, a TSS BED and ATACSeq BigWig can be paired to determine the area of the TSS that is most nucleosome-depleted—the most important feature for effective CRISPRi experiments.

### Identifying guide RNAs

The first processing module in CRISPRware is generate_guides, which scans an input genome for protospacers based on a user-defined PAM sequence. Any PAM sequence can be used as input and IUPAC ambiguity codes will be automatically expanded, e.g. --pam NGG is automatically expanded to --pam AGG,CGG,GGG,TGG and --pam TTTV is expanded to --pam TTTC,TTTG,TTTA. In cases where the pam is 5’ to the protospacer, such in Cas12A, the user should also use the --pam_5_prime argument. The length of the desired protospacer is passed with –-sgRNA_length [integer] with the default set to 20. GTF/GFF (hereafter GTF) and BED files can be passed to limit the search space to regions of interest. If a GTF is passed, a feature identifier (CDS, 5UTR, 3UTR, exon, etc.) can also be passed to further restrict the gRNAs discovered to only those for which the putative active site intersects the feature. Given a genomic fasta and a list of target sites, the search is limited to chromosomes that appear in the target list, and the search function can use multiple processes to reduce computation time. Additional options for filtering include poly-T or poly-G tracks, restriction enzyme sites, and GC percentage, with the complete list of arguments available on the Github repository. The user can also define a context window around the protospacer, which is a requirement for many downstream scoring methods. The default settings are for SpCas9 (NGG PAM, putative cleavage 4 bps upstream from the PAM) and Ruleset 3 scoring (30-mer target sequence centered on the protospacer).

### Building off-target index

Off-target scoring is performed by GuideScan2, and the wrapper script index_genome is used to generate the index from a fasta file. The wrapper script includes the option to subset the fasta to calculate the off-target effects against user-defined areas of interest (Fig. 2C, D).

### Scoring guide RNAs

Following gRNA identification, the output is passed to score_guides, which calculates the GuideScan2 off-target scoring and Ruleset 3 on-target scoring for each guide. Both scoring methods include multiprocessing, and the user can specify the number of protospacers to score concurrently to avoid excessive memory usage on large sets (Supp Fig. 1B). An arbitrary number of GuideScan2 indices can be passed, in which case each gRNA will be scored against each index.

If the user is interested in scoring Cas12a gRNAs or using an alternative scoring method for Cas9, the helper script crisprscore.R can be used. This script formats the input and applies an available scoring method from the crisprVerse Bioconductor package, adding a new column for each scoring method. Any number of scoring methods can be applied and filtered over. The output of crisprscore.R can then be passed to score_guides to reformat for the next module and for off-target scoring with Guidescan2 and/or Ruleset3 scoring. To disable Guidescan2 and/or Ruleset3 (i.e., simply reformat the data), the arguments --skip_gs2 and/or --skip_rs3, respectively, can be passed.

### Ranking guide RNAs

The final module, rank_guides, takes the scored guides as input and filters them according to user-defined criteria. Any number of scoring columns and minimum cut-offs can be used to filter out undesirable gRNAs. If the targets are genes represented by a GTF, the user can also specify the percentile range of the gene feature. For example, ‘-z-percentile_range 0 50 -feature CDS’ will filter out guides that are not within the first 50% of the coding sequence of any gene in the GTF. By default, min–max normalization is applied to scoring columns prior to standardizing scores before applying the final ranking.

### Model organism gRNA scoring

For the human genome sequence, GCA_000001405.15_GRCh38 was used, alternate haplotypes and mitochondrial chromosomes were removed, and pseudoautosomal Y regions hard masked (converted to Ns). Genome sequences hosted at the UCSC Genome Browser were obtained for model organisms, and alternate haplotypes, mitochondrial DNA, and Y chromosomes were discarded. For Cas9 gRNAs, generate_guides was run with default settings to find all gRNAs expected to cleave in the CDS of any NCBI RefSeq gene annotation (curated and predicted gene models). score_guides with Ruleset 3 scoring with both tracrRNA options (Hsu2013, Chen2013) was run. For DeepHF scoring, both the “T7” and “U6” options were run, and the Cas9 enzyme was set to “WT” in both cases. For other scoring methods, default settings were used.

### Full commands for model organism gRNA scoring

The commands we used to generate the model species tracks for the human genome are shown below. The same commands were run for other species with the appropriate genome fasta and RefSeq gene annotations.

####  Create Guidescan2 off-target index

index_genome -f hg38.fa

####  Create metagene gene models

preprocess_annotation -g hg38.gtf -m metagene

####  Find NGG-protospacers and score guides with Ruleset3

By default, any gRNA with 0, 1, or 2 mismatches to another protospacer is dropped, and specificity scores are calculated relative to all protospacers with 3 mismatches.

generate_guides -f hg38.fa -t 8 -k./annotations/meta_hg38.gtf score_guides -b sgRNAs/sgRNAs.bed -d --tracr both --threads 8 -i gscan_index/gscan_index

#### Finding NGG-protospacers and score guides with other methods

While Ruleset 1, Ruleset 2 (Azimuth), Ruleset 3, and DeepSpCas9 require a context length of 30 nts which are consistent with default parameters, DeepHF, CRISPRscan, and CRISPRater require contexts of 23, 35, and 20, respectively. In order to generate and score guides with these tools, we used:

generate_guides -o deephf -w 0 3 -f hg38.fa -t 8 -k./annotations/meta_hg38.gtf

crisprscore.R deephfsgRNAs.tsv 3 deephf_score.tsv WT U6

generate_guides -o crisprscan -w 6 9 -f hg38.fa -t 8 -k./annotations/meta_hg38.gtf

crisprscore.R crisprscansgRNAs.tsv 7 crisprscan_scored.tsv

generate_guides -o crisprater -w 0 0 -f hg38.fa -t 8 -k./annotations/meta_hg38.gtf

crisprscore.R crispratersgRNAs.tsv 10 crisprater_scored.tsv

#### Filter guides by score and position

After scoring with all tools, the files were merged. Finally, all guides were filtered for Guidescan2 off-target score > 0.2 and Ruleset 3 with Chen2013 tracrRNA > 0 and position in the 5 th-65 th percentile of the CDS.

rank_guides -k scoredsgRNAs.tsv -t annotations/meta_hg38.gtf

--feature CDS --filtering_columns specificity_gscan_index rs3_z_score_Chen2013 --minimum_values 0.2 0 --percentile_range 5 65

#### Finding TTTV-protospacers and score guides with other methods

Cas12A protospacer scoring is similar but has the following changes and the generate guides and scoring steps.

generate_guides -f hg38.fa -k./annotations/meta_hg38 --feature CDS --pam TTTV --sgRNA_length 23 -w 8 3 --pam_5_prime --active_site_offset_5 19 --active_site_offset_3 23 -t 6

crisprscore.R TTTVsgRNAs.tsv 5 DeepCpf1_scored_sgRNAs.tsv

crisprscore.R TTTVsgRNAs.tsv 6 EnPamGB_scored_sgRNAs.tsv

### Human cell line and mouse leukocyte consensus models

Transcript expression data was downloaded from the Encode consortium for each cell type using Encode’s uniform processing pipeline with Kallisto. Mapping is done against gencode.v29.annotation.gtf and gencode.vM21.annotation.gtf annotations for human and mouse, respectively. preprocess_annotation module was run in consensus mode with --min flag set to 0.1, 1, or 10.

### K562 transcription factor binding sites and off-target index

931 datasets were downloaded from the Encode K562 repository containing processed ChIP-Seq, ATAC-Seq, DNase-Seq, and ChIA-Pet in BED format. Hg19 datasets were removed, as were ChIP-Seq data indicating inactive genomic regions: H3K27me3 and HK9me3. This left 886 datasets (Supplementary Table 13). A K562 active genome was constructed by passing these BED files and the comprehensive Gencode v45 annotation to the index_genome module. The argument –-window 1000 1000 was used to expand the region around each active interval by 1000 bps in each direction to account for the local range of CRISPRi influence when calculating off-targets. For each transcription factor (TF) noted in Fig. 2B, IDR thresholded peaks in BED format were downloaded from Encode, and each was run through the CRISPRware pipeline with default settings. 

### Cast/C57 Hybrid Line

A C57BL/6 mouse was crossed to a Castaneous mouse (Cast/C57) to generate an F1 hybrid mouse gifted to the Carpenter lab line by the Anguera lab at the University of Pennsylvania. All mice were maintained at the Penn Vet animal facility. Experiments were approved by the University of Pennsylvania and the University of California Santa Cruz (UCSC), Institutional Animal Care and Use Committees (IACUC). Mice were sacrificed according to IACUC guidelines with CO_2_ followed by cervical dislocation, and legs were removed from a 12-week-old F1 mouse, shipped to the Carpenter lab on ice, and bone marrow was extracted as previously described [28]. Cells were grown in DMEM supplemented with 10% FCS, 5 ml pen/strep (100X), 500ul ciprofloxacin (10 mg/ml), and 10% L929 supernatant (which contains macrophage colony-stimulating factor) with the replacement of culture medium every 2 to 3 days. On day 3, cells were infected with CreJ2 virus to induce immortalization as previously described [28]. After 3 months, when cells were capable of doubling without supplementation with L929, they were deemed fully immortalized (iBMDM-Cast/C57). The iBMDM-Cast/C57 were then lentivirally infected with the dCas9-KRAB construct using Lenti-dCas9-KRAB-blast, addgene #89567. Cells were selected for knockdown efficiency greater than 90%.

### Allele-specific targeting

Castaneous SNPs were downloaded from the Mouse Genome Database [29] hosted at Mouse Genome Informatics [47]. A Castaneous genome was generated using CRISPRware library script

replace_snps.py GRCm39.primary_assembly.genome.fa Cast.genome.fa Castaneous_SNPs.txt

RNA-Seq from untreated BMDMs was taken from GSE120762 [48] and processed with Salmon using the Gencode v34 gene annotation to generate expression files for three replicates (BMDM_nt_1.sf, BMDM_nt_2.sf, BMDM_nt_3.sf)**.** CRISPRware was used to find windows around the TSSs of transcribed isoforms expressed at TPM > 10 in each replicate. CRISPRi has superior performance when slightly downstream of the TSS, so a window of + 0 to + 300 was used.

preprocess_annotation --gtf gencode.vM34.annotation.gtf --tpm_files BMDM_nt_1.sf,BMDM_nt_2.sf,BMDM_nt_3.sf --min 10 --tss_window 0 300

C57 ATAC-Seq peaks in resting BMDMs were downloaded from GSE109965 [49], and coordinates were lifted from mm10 to mm39 mouse genome build. Protospacers were obtained for both C57 and Cast genomes such that they intersected with the TSS window and open ATAC peaks with the following commands.

generate_guides \

--fasta GRCm39.primary_assembly.genome.fa \

--active_site_offset_5=−20 \

--active_site_offset_3=0 \

--locations_to_keep C57_ATAC.bed TSS_gencode.vM34.annotation.bed \

--output_prefix C57_gRNAs

generate_guides \

--fasta Cast.genome.fa \

--active_site_offset_5=−20 \

--active_site_offset_3=0 \

--locations_to_keep C57_ATAC.bed TSS_gencode.vM34.annotation.bed \

--output_prefix Cast_gRNAs

Sequences from both outputs were then scored using the score_guides module with default parameters. Scored gRNAs were compared with a CRISPRware library script, with minimum hamming distance set to “1”,

allele_specific_guides.py C57_gRNAs.tsv Cast_gRNAs.tsv -d 1

This script produces three outputs: C57 specific protospacers and Castaneous specific protospacers, which indicate a lost or gained PAM, and protospacers where the PAM is maintained but there is at least one SNP in the protospacer. We focused on protospacers where the PAM is lost or gained in Cast relative to C57 and filtered for high predicted on-target activity from Ruleset 3 scoring (score > 1) and low off-target effects (Guidescan2 score > 0.2). We then filtered genes to find those that could be targeted with allele-specific guides and which had SNPs in the coding sequence so that we could verify knockdown by sequencing RNA. We manually examined the genes in the UCSC Genome Browser [28] and verified that the gene Eipr1 met all criteria and, uniquely, had three Cast specific SNPs in close proximity in exon 8. The SNPs were in the coding sequence of the primary isoform, which we expected to give us high confidence in assigning RNA reads to the strain-specific chromosome. After infection with gRNAs and selection, we collected RNA and generated cDNA. We used primers Eipr1_F TGTGGAGTGTGCGCTACAAC and Eipr1_R ACCAGTCTCCCGTCGTAACT to amplify the region around the SNPs and had the sample Amplicon-Sequenced. Sequence reads were mapped with STAR, and SNP abundance was quantified using BCFTools.

## Supplementary Information

Supplementary Material 1.

Supplementary Material 2.

## Data Availability

CRISPRware code and documentation are maintained at https://github.com/ericmalekos/crisprware. Genome browser sessions for NGG and TTTV gRNA libraries are stably hosted for all six model species: • Hg38: https://www.genome.ucsc.edu/s/CarpenterLab/crisprware_hg38. • Mm39: https://genome.ucsc.edu/s/CarpenterLab/crisprware_mm39. • Rn7: https://genome.ucsc.edu/s/CarpenterLab/crisprware_rn7.. • DanRer11: https://genome.ucsc.edu/s/CarpenterLab/crisprware_danRer11. • Dm6: https://genome.ucsc.edu/s/CarpenterLab/crisprware_dm6. • Ce11: https://genome.ucsc.edu/s/CarpenterLab/crisprware_ce11. Project name: CRISPRware. Project home page: https://github.com/ericmalekos/crisprware. Operating system(s): MacOS, Ubuntu, Docker. Programming language: Python. Other requirements: conda package manager or Docker. License: GNU GPL. Any restrictions to use by non-academics: license needed.

## Abbreviations

gRNA: Guide RNA
NGS: Next generation sequencing
CDS: Coding sequence
TPM: Transcripts per million
CRISPRi: CRISPR interference
CRISPRa: CRISPR activation
Cast: Castaneous mouse strain
