# Supplementary material for: CRISPRware: a software package for contextual gRNA library design
Source: BMC Genomics. 2025 Jul 1;26:607. doi: 10.1186/s12864-025-11775-8 (PMC12210694; doi:10.1186/s12864-025-11775-8)
Supplement: Supplementary file 2 — Supplementary Material 2. [file 12864_2025_11775_MOESM2_ESM.pdf]

A

	Coding genes	Coding transcripts	TTTV CDS protospacers	5th to 65th % CDS	DeepCpf1 > 0.5	gRNA/ gene	EnPAMGB > 0.5	gRNA/ gene
**Hg38**	20,627	139,092	1,028,363	496,119	321,043	9	633,331	16
**Rn7**	22,228	74,754	1,045,523	517,760	446,590	10	517,760	18
**Mm39**	22,864	98,005	1,071,685	529,667	457,223	10	529,667	18
**DanRer11**	26,424	57,093	1,285,350	607,824	543,521	11	607,824	21
**Dm6**	13,968	30,717	566,165	277,448	231,203	8	277,448	16
**Ce11**	20,093	28,146	1,173,706	582,607	358,067	15	582,607	29

B

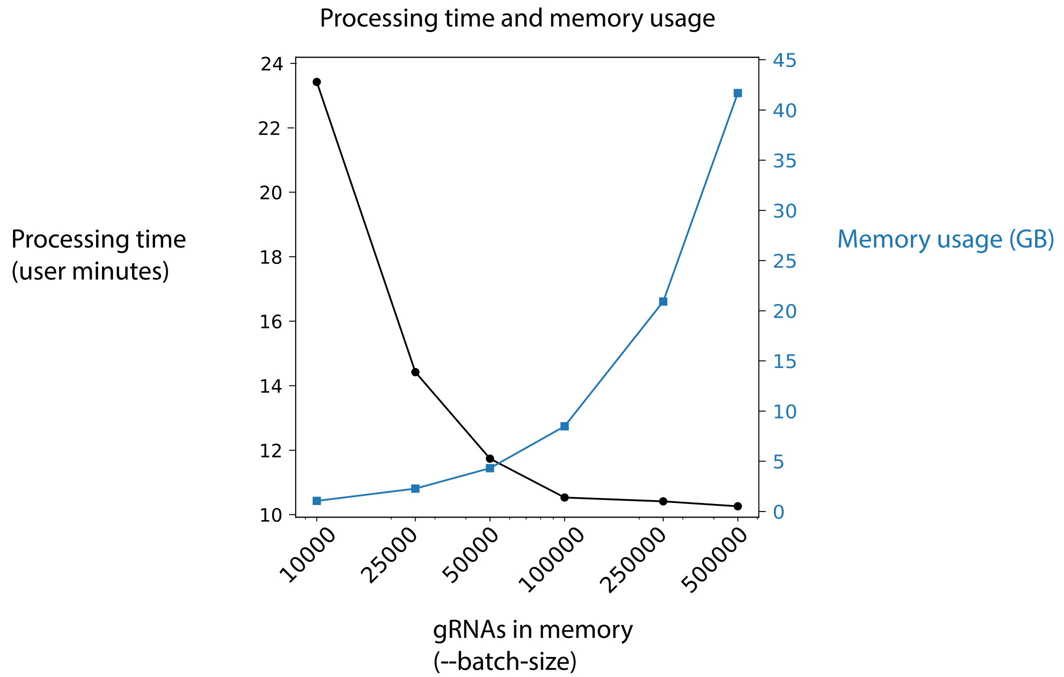

Supplementary Figure 1 (A) Demonstration of CRISPRware applied on model genomes with NCBI RefSeq gene annotations, targeting TTTV PAMs. Scores are determined by DeepCpf1 for AsCas12A and enPAMGB for enCas12a as implemented in crisperVerse. After positional filtering (5th-65th percentile), subsequent filtering is not applied sequentially, and gRNA/gene reports the median available gRNA under each filtering criteria. **(B)** Running enPAMGB scoring on 500,000 gRNAs at various batch sizes. Reducing batch size decreases memory requirements while slightly increasing running time.

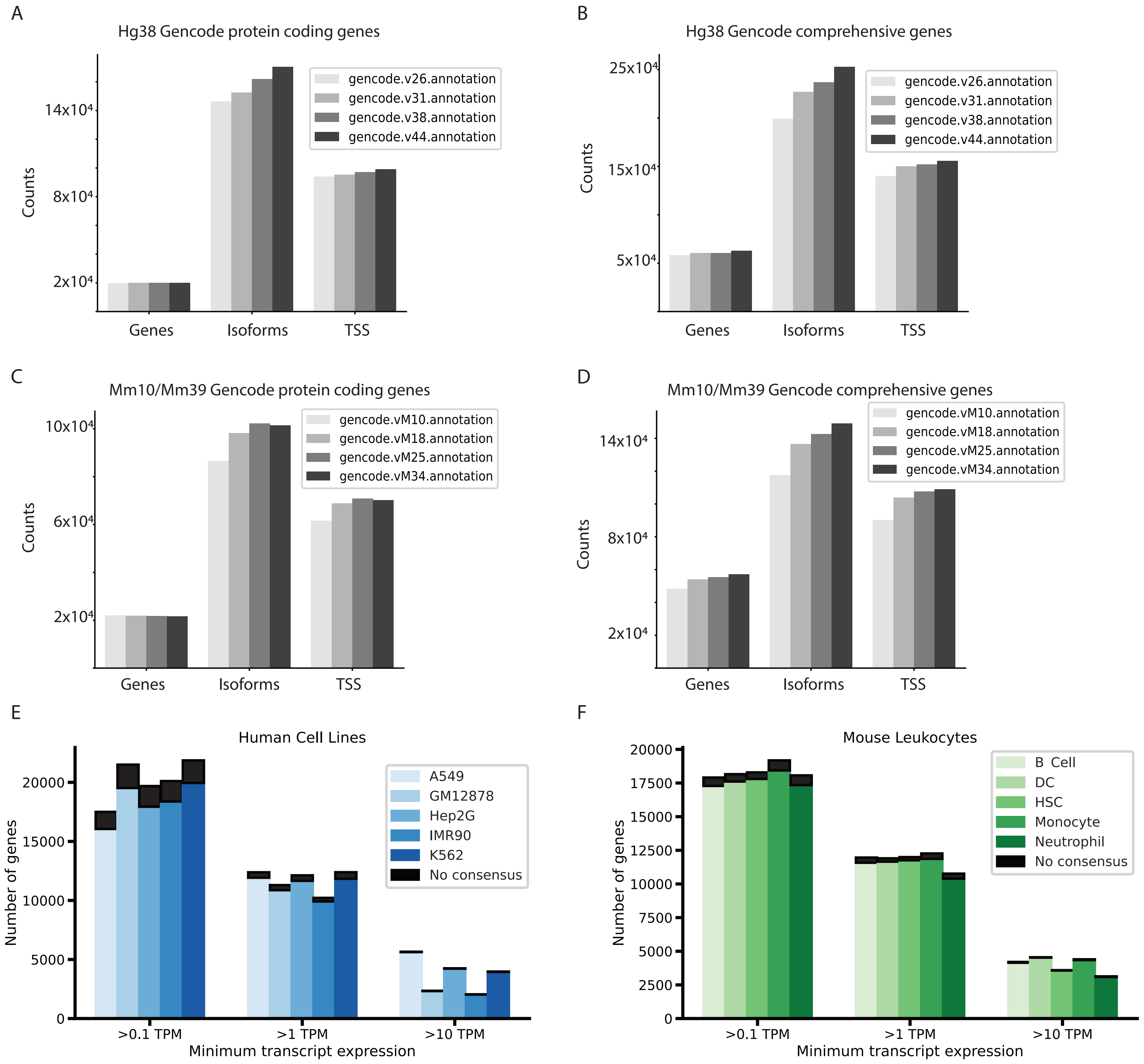
A
Hg38 Gencode protein coding genes
Counts
14x10⁴
8x10⁴
2x10⁴
Genes
Isoforms
TSS
gencode.v26.annotation
gencode.v31.annotation
gencode.v38.annotation
gencode.v44.annotation
B
Hg38 Gencode comprehensive genes
Counts
25x10⁴
15x10⁴
5x10⁴
Genes
Isoforms
TSS
gencode.v26.annotation
gencode.v31.annotation
gencode.v38.annotation
gencode.v44.annotation
C
Mm10/Mm39 Gencode protein coding genes
Counts
10x10⁴
6x10⁴
2x10⁴
Genes
Isoforms
TSS
gencode.vM10.annotation
gencode.vM18.annotation
gencode.vM25.annotation
gencode.vM34.annotation
D
Mm10/Mm39 Gencode comprehensive genes
Counts
14x10⁴
8x10⁴
2x10⁴
Genes
Isoforms
TSS
gencode.vM10.annotation
gencode.vM18.annotation
gencode.vM25.annotation
gencode.vM34.annotation
E
Human Cell Lines
Number of genes
20000
15000
10000
5000
0
>0.1 TPM
>1 TPM
>10 TPM
Minimum transcript expression
A549
GM12878
Hep2G
IMR90
K562
No consensus
F
Mouse Leukocytes
Number of genes
20000
17500
15000
12500
10000
7500
5000
2500
0
>0.1 TPM
>1 TPM
>10 TPM
Minimum transcript expression
B Cell
DC
HSC
Monocyte
Neutrophil
No consensus

Supplementary Figure 2 (A-D) Changes in the number of protein-coding genes, protein-coding gene isoforms, and protein-coding isoform TSSs **(A, C)** and from the comprehensive protein-coding and noncoding Gencode releases and **(B, D)**. **(E, F)** The number of expressed genes for which consensus gene models can be constructed at increasingly stringent expression cutoffs.

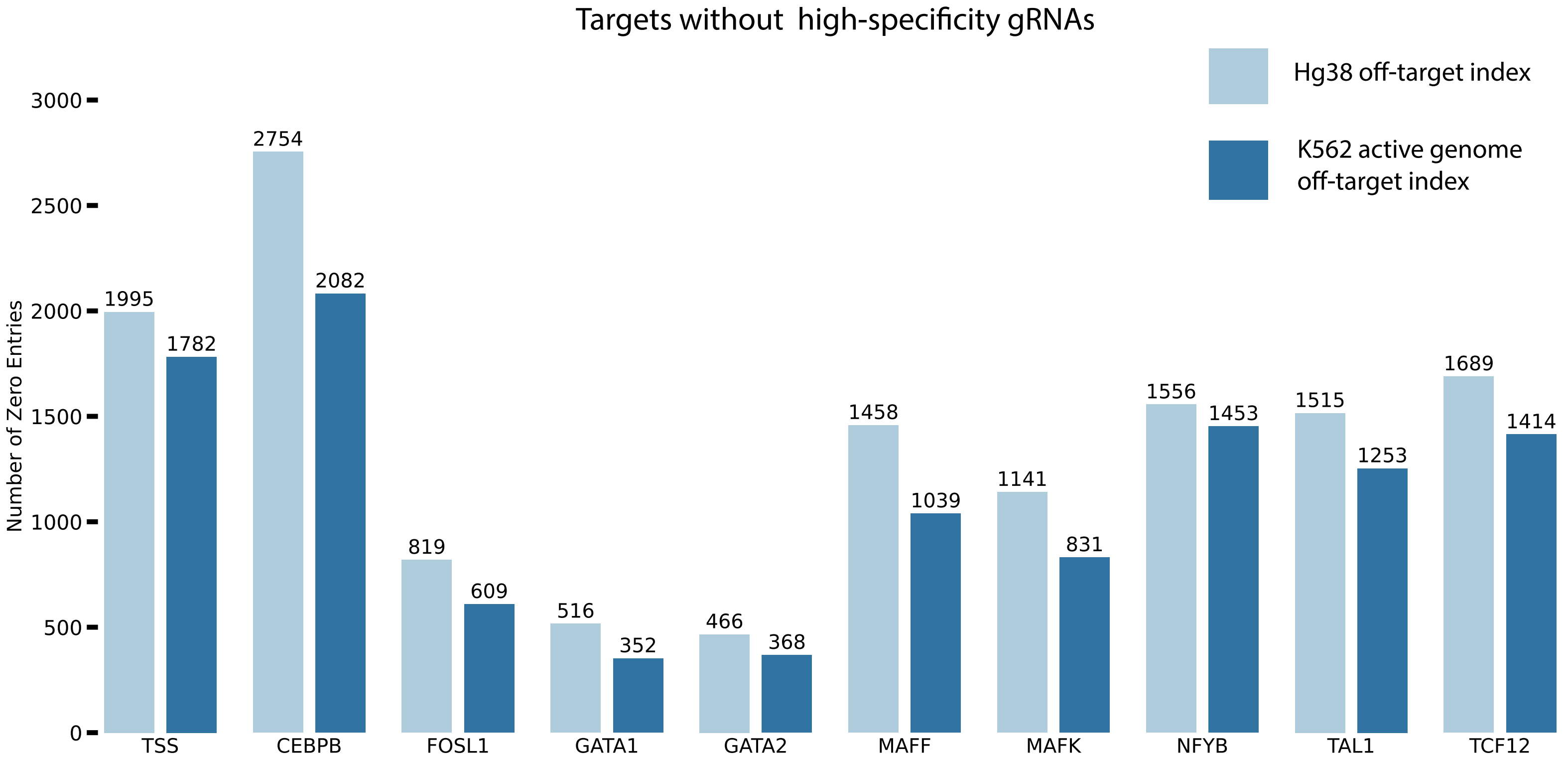
Targets without high-specificity gRNAs
Hg38 off-target index
K562 active genome off-target index
Number of Zero Entries
3000
2500
2000
1500
1000
500
0
1995
1782
2754
2082
819
609
516
352
466
368
1458
1039
1141
831
1556
1453
1515
1253
1689
1414
TSS
CEBPB
FOSL1
GATA1
GATA2
MAFF
MAFK
NFYB
TAL1
TCF12

Supplementary Figure 3 (A) Number of target sites, either TSS or transcription factor binding sites, for which there are 0 high specificity gRNAs (GuideScan2 score > 0.2, > 2 mismatches) against either the entire Hg38 genome or the active portions of the genome in K562. See Methods for construction of K562 active genome.

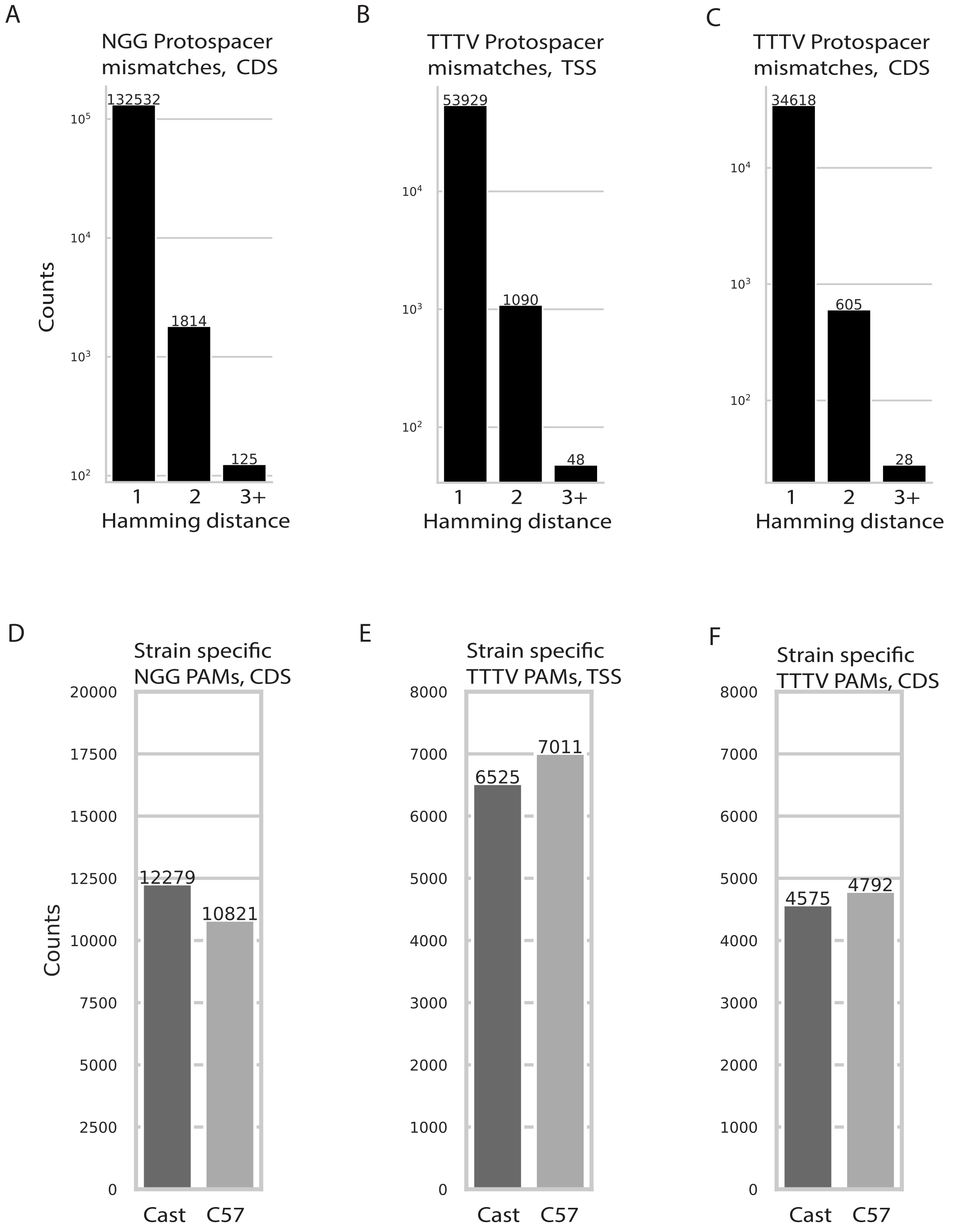
A
NGG Protospacer mismatches, CDS
Counts
10^5
10^4
10^3
10^2
132532
1814
125
1
2
3+
Hamming distance
B
TTTV Protospacer mismatches, TSS
10^4
10^3
10^2
53929
1090
48
1
2
3+
Hamming distance
C
TTTV Protospacer mismatches, CDS
10^4
10^3
10^2
34618
605
28
1
2
3+
Hamming distance
D
Strain specific NGG PAMs, CDS
Counts
20000
17500
15000
12500
10000
7500
5000
2500
0
12279
10821
Cast
C57
E
Strain specific TTTV PAMs, TSS
8000
7000
6000
5000
4000
3000
2000
1000
0
6525
7011
Cast
C57
F
Strain specific TTTV PAMs, CDS
8000
7000
6000
5000
4000
3000
2000
1000
0
4575
4792
Cast
C57

Supplementary Figure 4 (A-C) Mismatches in the protospacer sequence between C57BL/6 and Castaneous in CDS regions with NGG pam **(A)**, the TSS with TTTV PAMs **(B)**, and the CDS with TTTV PAMs **(C)**. **(D-F)** Lost/gained PAMs in Castaneous vs. C57BL/6.

TSS-targeting is defined as a +/- 300 bp window around a Gencode vM34 TSS.
